# Effects of low doses of esmolol on cardiac and vascular function in experimental septic shock

**DOI:** 10.1186/s13054-016-1580-2

**Published:** 2016-12-21

**Authors:** Chaojie Wei, Huguette Louis, Margaux Schmitt, Eliane Albuisson, Sophie Orlowski, Bruno Levy, Antoine Kimmoun

**Affiliations:** 1 INSERM U 1116, Groupe Choc, Equipe 2, Faculté de Médecine, Vandoeuvre les Nancy, France; 2 Université de Lorraine, Nancy, France; 3 INSERM U 1116, Groupe Choc, Equipe 1, Faculté de Médecine, Vandoeuvre les Nancy, France; 4 Unité ESPRI-BioBase, CHRU Nancy, Vandoeuvre les Nancy, France; 5 CHU Nancy, Service de Réanimation Médicale Brabois, Pole Cardiovasculaire et Réanimation Médicale, Hôpital Brabois, Vandoeuvre les Nancy, France

**Keywords:** Sepsis, Esmolol, Heart function, Vasoreactivity, Inflammation

## Abstract

**Background:**

Administration of a selective β1-blocker, such as esmolol, in human septic shock has demonstrated cardiovascular protective effects related to heart rate reduction. Certain experimental data also indicate that esmolol exerts systemic anti-inflammatory and beneficial effects on vascular tone. Thus, the present study aimed to determine whether a non-chronotropic dose of esmolol maintains its protective cardiovascular and anti-inflammatory effects in experimental septic shock.

**Methods:**

Four hours after cecal ligation and puncture (CLP), Wistar male rats were randomly allocated to the following groups (*n* = 8): CLP, CLP + E-1 (esmolol: 1 mg.kg^−1^.h^−1^), CLP + E-5 (esmolol: 5 mg.kg^−1^.h^−1^), CLP + E-18 (esmolol: 18 mg.kg^−1^.h^−1^). An additional eight rats underwent sham operation. All rats received a continuous infusion of saline, analgesic and antibiotics 4 hours after the surgery. Assessment at 18 hours included in vivo cardiac function assessed by echocardiography and ex vivo vasoreactivity assessed by myography. Circulating cytokine levels (IL-6 and IL-10) were measured by ELISA. Cardiac and vascular protein expressions of p-NF-κB, IκBα, iNOS, p-AKT/AKT and p-eNOS/eNOS were assessed by western blotting.

**Results:**

CLP induced tachycardia, hypotension, cardiac output reduction, hyperlactatemia and vascular hypo-responsiveness to vasopressors. Compared to CLP animals, heart rate was unchanged in CLP + E-1 and CLP + E-5 but was reduced in CLP + E-18. Stroke volume, cardiac output, mean arterial pressure and lactatemia were improved in CLP + E-1 and CLP + E-5, while vascular responsiveness to phenylephrine was only improved in CLP + E-5 and CLP + E-18. Plasma IL-6 levels were decreased in all esmolol groups. p-NF-κB was decreased in both cardiac and vascular tissues in CLP + E-5 and CLP + E-18.

**Conclusion:**

In experimental septic shock, low doses of esmolol still improved cardiac function and vasoreactivity. These benefits appear to be associated with a modulation of inflammatory pathways.

**Electronic supplementary material:**

The online version of this article (doi:10.1186/s13054-016-1580-2) contains supplementary material, which is available to authorized users.

## Background

Septic shock is associated with dysfunction of the autonomic system triggered by a massive release of pro-inflammatory cytokines [1–4]. Loss of cardiovascular variability, inappropriate tachycardia and excessive catecholamine release leading to the onset of multiple organ failure and death are the main symptoms of this autonomic dysfunction. Selective β1-blockade is one approach currently under assessment to downregulate this dysfunction [5–7].

In animals with experimental septic shock, a selective β1-blocker such as esmolol efficiently improves both cardiac and vascular functions [8–13]. Most of these hemodynamic changes could be related to the pleiotropic effects of esmolol, including reduction in heart rate, but also to downregulation of inflammatory pathways. Nevertheless, reduction in heart rate alone does not explain all of the beneficial effects observed with esmolol. Indeed, we previously demonstrated in an experimental model of septic shock that isolated reduction in heart rate by ivabradine (an *I*_*f*_ channel inhibitor) does not induce any change in inflammatory status [14].

In humans, Morelli, et al. and others have demonstrated in their respective studies that esmolol is efficient in decreasing heart rate in patients with septic shock [10, 15, 16]. Morelli et al. also observed a reduction in norepinephrine requirement in treated patients [10, 16]. In these studies, the dose of esmolol was chosen to achieve a reduction in heart rate of approximately 20%. This dose, however, was systematically associated with an initial decrease in cardiac output, thus rendering its prescription delicate in hemodynamically precarious patients with sepsis. On the other hand, Ibrahim-Zada et al. demonstrated that low-dose esmolol (6.7 μg.kg^−1^.min^−1^), which does not reduce heart rate, increases the survival rate in an endotoxic mouse model of sepsis. The authors hypothesize that the survival benefits are related to the modulation of inflammatory genes [17].

Using an experimental model of septic shock, the purpose of the present study was to determine whether low doses of esmolol, which do not reduce heart rate, are associated with beneficial effects on: (1) in vivo left ventricular systolic function assessed by echocardiography, (2) ex vivo vascular responsiveness to phenylephrine (Phe) and vascular relaxation to acetylcholine (Ach) of the thoracic aorta and mesenteric artery and (3) systemic, cardiac and vascular inflammatory pathways.

## Method

### Animals

Male Wistar rats (Janvier, France) weighing 300–400 g were used after an acclimation period of at least one week prior to experimentation. All animal experimentation protocols were approved by the French Animal Care Committee in keeping with European regulations. The study was conducted in a university research laboratory.

### Shock model: cecal ligation and puncture (CLP)

This model has previously been described in detail [14]. Additional information on this model is provided in Additional file 1.

### Fluid resuscitation management

Four hours after CLP induction, all rats received a continuous infusion of saline, analgesic (nalbuphin 0.2 mg.kg^−1^.h^−1^) and anti-infective therapy (imipenem and cilastatin sodium 10 mg.kg^−1^). The total infusion rate was fixed at 10 ml.kg^−1^.h^−1^.

### Esmolol dosage

The aim of this pre-experimental series was to identify a dose of esmolol that does not reduce heart rate 18 hours after the onset of CLP. Given that esmolol at 10 mg.kg^−1^.h^−1^ still induced reduction in heart rate at 18 hours after onset of CLP in the study of Suzuki et al., the first tested dose was 5 mg.kg^−1^.h^−1^ (*n* = 4), initiated 4 hours after surgery [8]. A second group also received esmolol at 18 mg.kg^−1^.h^−1^ (*n* = 4). Heart rate in these two groups was compared with heart rate in a CLP group (*n* = 8) extracted from our previous study using the exact same protocol [14]. Heart rate was calculated in each group by echocardiography at 18 hours after the onset of CLP. Infusion of esmolol 5 mg.kg^−1^.h^−1^ did not reduce heart rate compared to heart rate in the CLP group (Additional file 2: Figure S1). In view of the hypothesis of Ibrahim-Zada et al. suggesting that very low doses of selective β1-adrenoreceptor antagonist still present immunomodulatory effects in endotoxic shock, an esmolol dose of 1 mg.kg^−1^.h^−1^ was also tested [17].

### Experimental design

Four hours after CLP, Wistar rats were randomly allocated to one of the following groups: CLP (*n* = 8), CLP + E-1 (esmolol 1 mg.kg^−1^.h^−1^ initiated 4 hours after surgery for a period of 14 hours (*n* = 8)), CLP + E-5 (esmolol 5 mg.kg^−1^.h^−1^ initiated 4 hours after surgery for a period of 14 hours (*n* = 8)), or CLP + E-18 (esmolol 18 mg.kg^−1^.h^−1^ initiated 4 hours after surgery for a period of 14 hours (*n* = 8)). Rats that underwent sham operation were allocated to the sham group. Assessments were performed 18 hours after surgery.

### Echocardiography procedure

Hemodynamic investigation was initiated 18 hours after surgery. This procedure has previously been detailed and is provided in Additional file 1 [14].

### Vascular reactivity procedure

After the animals were killed by exsanguination under isoflurane, the thoracic aorta and mesentery were removed and their vasoreactivity was studied. The procedure is detailed in Additional file 1.

### Cytokine analyses

Plasma IL-6 and IL-10 were measured in duplicate with the use of rat IL-6 and IL-10 ELISA kits (Quantikine ELISA; R&D Systems Europe, Abingdon, UK) according to the manufacturer’s instructions. Results are expressed as picograms of the measured cytokine per milliliter of plasma.

### Western blotting

The thoracic aorta and heart were homogenized and lysed, and proteins (25 μg) were separated on 10% SDS-PAGE. Blots were probed with the following antibodies: phosphorylated-AKT (p-AKT) (rabbit anti-rat AKT, phosphorylated (Ser473)), AKT (AKT-pan (C67E7)), phosphorylated endothelial nitric oxide synthase (p-eNOS) (rabbit anti-rat eNOS, phosphorylated (Ser1177)), endothelial nitric oxide synthase (eNOS) (rabbit anti-rat eNOS), phosphorylated NF-κB (Phospho-NF-κB p65 (Ser536) (93H1)) and IκBα ((44D4)), all from Cell Signaling Technology, Saint Quentin Yvelines, France; anti-iNOS (Abcam, Paris, France) and β-actin (13E5) (Cell Signaling Technology, Saint Quentin Yvelines, France). Proteins were transferred onto nitrocellulose membranes after which bound antibodies were detected with a secondary peroxidase-conjugated anti-rabbit IgG (Promega, Madison, WI, USA). The blots were visualized using an enhanced chemiluminescence system (ECL Plus; Amersham, GE Healthcare Europe, Velizy-Villacoublay, France).

### RNA extraction and quantitative reverse transcriptase-polymerase chain reaction of α1-adrenoreceptor, β1-adrenoreceptor and β2-adrenoreceptor

Total RNA extraction was carried out with the RNA Plus mini Kit (Qiagen, Courtaboeuf Cedex, France) according to the manufacturer’s instructions. The procedure is provided in Additional file 1.

### Statistical analysis

Results are expressed as median with minimum and maximum values in the main text, as median with minimum, maximum values and interquartile range (median (min-max); (interquartile range IQR)) in the tables, and as median with upper edges of error bars representing the 75th percentile in the figures. Because of the small sample size, all analyses were performed with nonparametric methods. The Mann–Whitney *U* test was used to determine whether the two independent samples in the sham and CLP groups stemmed from a population with a common median. The Kruskal-Wallis test was used to determine whether the four independent samples in the CLP, CLP + E-1, CLP + E-5 and CLP + E-18 groups stemmed from a population with a common median. When the Kruskal-Wallis test was significant at the 5% level, post hoc comparisons were performed between the CLP, CLP + E-1, CLP + E-5 and CLP + E-18 groups, using Dunn’s multiple comparisons procedure. The PRISM nonlinear curve-fitting tool (GraphPad Software, San Diego, CA, USA) was used to depict the results of vasoreactivity testing. The two-tailed statistical significance level was set at 5%. Statistical analyses were performed using IBM-SPSS Statistics 22.0 (IBM corp.) and GraphPad Software 6.0, (San Diego, CA, USA).

## Results

### Model characterization

Compared to animals in the sham group, CLP induced arterial hypotension (sham 103 mmHg (94–114); CLP 74 mmHg (59–83)) and hyperlactatemia (sham 1.1 mmol.l^−1^ (0.9–1.9); CLP 2.5 mmol.l^−1^ (1.6–3.5)). Cardiac output was decreased with CLP compared to animals in the sham group (sham: 111 ml.min^−1^ (94–122); CLP: 43 ml.min^−1^ (25–58)) (Table 1).

**Table 1:** Comparison of hemodynamic characteristics 18 hours after cecal ligation and puncture using echocardiography

Variables	Sham		IQR	CLP		IQR	*P* value
	*n* = 8			*n* = 8			
	Median	Min-Max		Median	Min-Max		
Heart rate (min^−1^)	372	361–396	368–373	419	349–472	381–452	0.015
Mean arterial pressure (mmHg)	103	94–114	100–113	74	59–83	67–81	<0.001
LVIDs (mm)	3.85	3.60–4.63	3.78–4.22	2.76	0.99–4.07	2.26–3.46	0.003
LVIDd (mm)	7.66	7.33–8.51	7.41–8.23	5.41	2.70–7.38	4.99–6.24	<0.001
Stroke volume (μl)	302	256–327	277–304	92	71–144	73–120	<0.001
Cardiac output (ml.min^−1^)	111	94–122	107–113	43	25–58	29–49	<0.001
Ejection fraction (%)	85	82–88	83–87	88	51–94	84–92	0.083
Lactatemia (mmol.l^−1^)	1.1	0.9–1.9	1.0–1.5	2.5	1.6–3.5	2.1–2.8	<0.001

Data were analyzed using the Mann–Whitney *U* test. *CLP* cecal ligature and puncture, *LVIDs* left ventricular internal diameter end systole, *LVIDd* left ventricular internal diameter end diastole

**Fig. 1:**
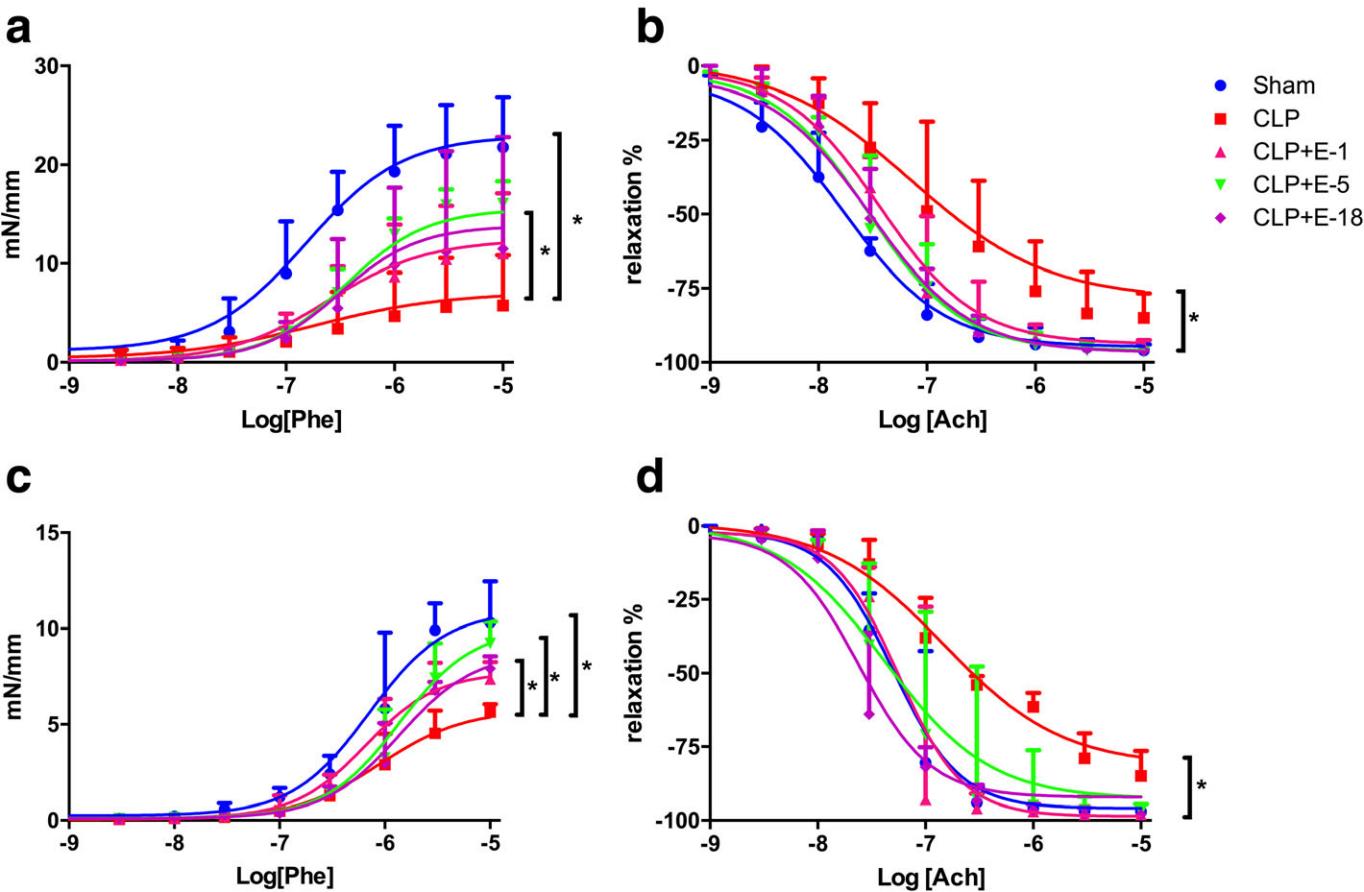
Vasoreactivity evaluated by myography. Ex vivo vascular responsiveness to phenylephrine and concentration–response curves to acetylcholine in rat thoracic aorta and mesenteric resistance arteries from sham, cecal ligation and puncture (*CLP*) and CLP + esmolol (*CLP* + *E*) groups (*n* = 8 per group). **a** and **c** Contraction of the vessel (in mN.mm^−1^) as a function of increasing concentrations of phenylephrine (*Phe*) expressed as log of Phe [M, mole/L]. **b** and **d** Relaxation of the vessel (in percent) as a function of increasing concentrations of acetylcholine (*Ach*) expressed as log of Ach [M, mole/L]. C*ircles*, *squares* and *triangles* represent median; *upper edges* of *error bars* represent the 75th percentile in each group; *p < 0.05. *Sham vs. CLP, CLP vs. CLP + E-5 and CLP vs. CLP + E-18 (**b**). *Sham vs. CLP, CLP vs. CLP + E-1 and CLP vs. CLP + E-18 (**d**). *E*-*1* esmolol 1 mg.kg^−1^.h^−1^, *E*-*5* esmolol 5 mg.kg^−1^.h^−1^, *E*-*18* esmolol 18 mg.kg^−1^.h^−1^. **a** and **b** aorta and **c** and **d** mesenteric arteries

In the isolated aorta and small mesenteric arteries (SMA), compared to animals in the sham group, CLP conversely blunted Phe-induced contraction (Fig. 1a, c). Compared to animals in the sham group, vascular responses to Ach were decreased with CLP in the isolated aorta and SMA (Fig. 1b, d).

### Hemodynamic effects after different doses of esmolol in animals that underwent CLP

Addition of esmolol at 18 mg.kg^−1^.h^−1^ induced a decrease in heart rate in the CLP group (CLP 419 bpm (349–472); CLP + E-18 366 bpm (342–383)), whereas infusion of 1 or 5 mg.kg^−1^.h^−1^ of esmolol did not induce reduction in heart rate (CLP 419 bpm (349–472); CLP + E-1 429 bpm (392–456); CLP + E-5 419 bpm (396–434)). Addition of esmolol at 5 mg.kg^−1^.h^−1^ or 1 mg.kg^−1^.h^−1^ increased cardiac output (CLP 43 ml.min^−1^ (25–58); CLP + E-1 86 ml.min^−1^ (66–97); CLP + E-5 89 ml.min^−1^ (66–101); CLP + E-18 60 ml.min^−1^ (49–80)), but decreased hyperlactatemia (CLP 2.5 mmol.l^−1^ (1.6–3.5); CLP + E-1 1.3 mmol.l^−1^ (0.7–1.5); CLP + E-5 1.4 mmol.l^−1^ (0.6–2.0); CLP + E-18 1.6 mmol.l^−1^ (1.2–2.4)) (Table 2).

**Table 2:** Comparison of hemodynamic characteristics 18 hours after cecal ligation and puncture using echocardiography

Variables	CLP		IQR	CLP + E-1		IQR	CLP + E-5		IQR	CLP + E-18		IQR	*P* value	Post hoc analysis
	*n* = 8			*n* = 8			*n* = 8			*n* = 8				
	Median	Min–Max		Median	Min–Max		Median	Min–Max		Median	Min–Max			
Heart rate (min^−1^)	419	349–472	381–452	429	392–456	414–436	419	396–434	402–428	366	342–383	354–376	0.002	1.000: CLP vs. CLP + E-1
														1.000: CLP vs. CLP + E-5
														0.019: CLP vs. CLP + E-18
Mean arterial pressure (mmHg)	74	59–83	67–81	89	86–92	88–90	90	77–97	85–95	82	71–114	78–87	0.001	0.002: CLP vs. CLP + E-1
														0.005: CLP vs. CLP + E-5
														0.624: CLP vs. CLP + E-18
LVIDs (mm)	2.76	0.99–4.07	2.26–3.46	3.35	2.75–3.77	3.00–3.73	3.67	2.89–3.97	3.10–3.93	3.21	2.04–3.97	2.98–3.33	0.118	
LVIDd (mm)	5.41	2.70–7.38	4.99–6.24	6.86	6.13–7.33	6.63–7.23	7.14	6.10–7.88	6.58–7.68	6.64	5.86–7.38	6.38–6.79	0.008	0.090: CLP vs. CLP + E-1
														0.012: CLP vs. CLP + E-5
														0.679: CLP vs. CLP + E-18
Stroke volume (μl)	92	71–144	73–120	207	153–225	172–220	215	154–238	183–234	160	142–215	148–209	<0.001	0.002: CLP vs. CLP + E-1
														<0.001: CLP vs. CLP + E-5
														0.092: CLP vs. CLP + E-18
Cardiac output (ml.min^−1^)	43	25–58	29–49	86	66–97	76–94	89	66–101	74–97	60	49–80	53–75	<0.001	<0.001: CLP vs. CLP + E-1
														<0.001: CLP vs. CLP + E-5
														0.528: CLP vs. CLP + E-18
Ejection fraction (%)	88	51–94	84–92	86	83–92	85–90	86	83–89	84–88	87	83–95	86–89	0.685	
Lactatemia (mmol.l^−1^)	2.5	1.6–3.5	2.1–2.8	1.3	0.7–1.5	1.2–1.4	1.4	0.6–2.0	1.0–1.5	1.6	1.2–2.4	1.3–1.9	0.001	0.001: CLP vs. CLP + E-1
														0.007: CLP vs. CLP + E-5
														0.128: CLP vs. CLP + E-18

Data were analyzed using the Kruskal-Wallis test. When significant at the 5% level, post hoc comparisons were performed between CLP and CLP + E-1, CLP + E-5, CLP + E-18 groups using Dunn’s multiple-comparisons test. *CLP* cecal ligation and puncture, *Min*–*Max* minimum–maximum, *IQR* interquartile range, *CLP* + *E*-*1* CLP + esmolol at 1 mg.kg^−1^.h^−1^, *CLP* + *E*-*5* CLP + esmolol at 5 mg.kg^−1^.h^−1^, *CLP* + *E*-*18* CLP + esmolol at 18 mg.kg^−1^.h^−1^, *LVIDs* left ventricular internal diameter end systole, *LVIDd* left ventricular internal diameter end diastole

Compared to animals in the CLP group, maximum contractility of the thoracic aorta to Phe tended to increase under esmolol infused at all tested doses, although only the increase with 5 mg.kg^−1^.h^−1^ of infused esmolol was statistically significant (Fig. 1a). Maximum relaxation ability of the thoracic aorta to Ach tended to be improved by esmolol at all doses although it was only statistically significant at 5 and 18 mg.kg^−1^.h^−1^ (Fig. 1b).

Compared to animals in the CLP group, the maximum contractility of SMA to Phe also tended to increase under esmolol at all doses. Statistical significance was only observed under esmolol infused at 5 and 18 mg.kg^−1^.h^−1^ (Fig. 1c). Maximum relaxation ability of SMA to Ach tended to be improved by esmolol at all doses. Esmolol infused at 1 and 18 mg.kg^−1^.h^−1^ (Fig. 1d) had a statistically significant effect, whereas esmolol at 5 mg.kg^−1^.h^−1^ did not demonstrate significant improvement (Fig. 1d).

### Effect of different doses of esmolol on circulatory inflammatory mediators

**Fig. 2:**
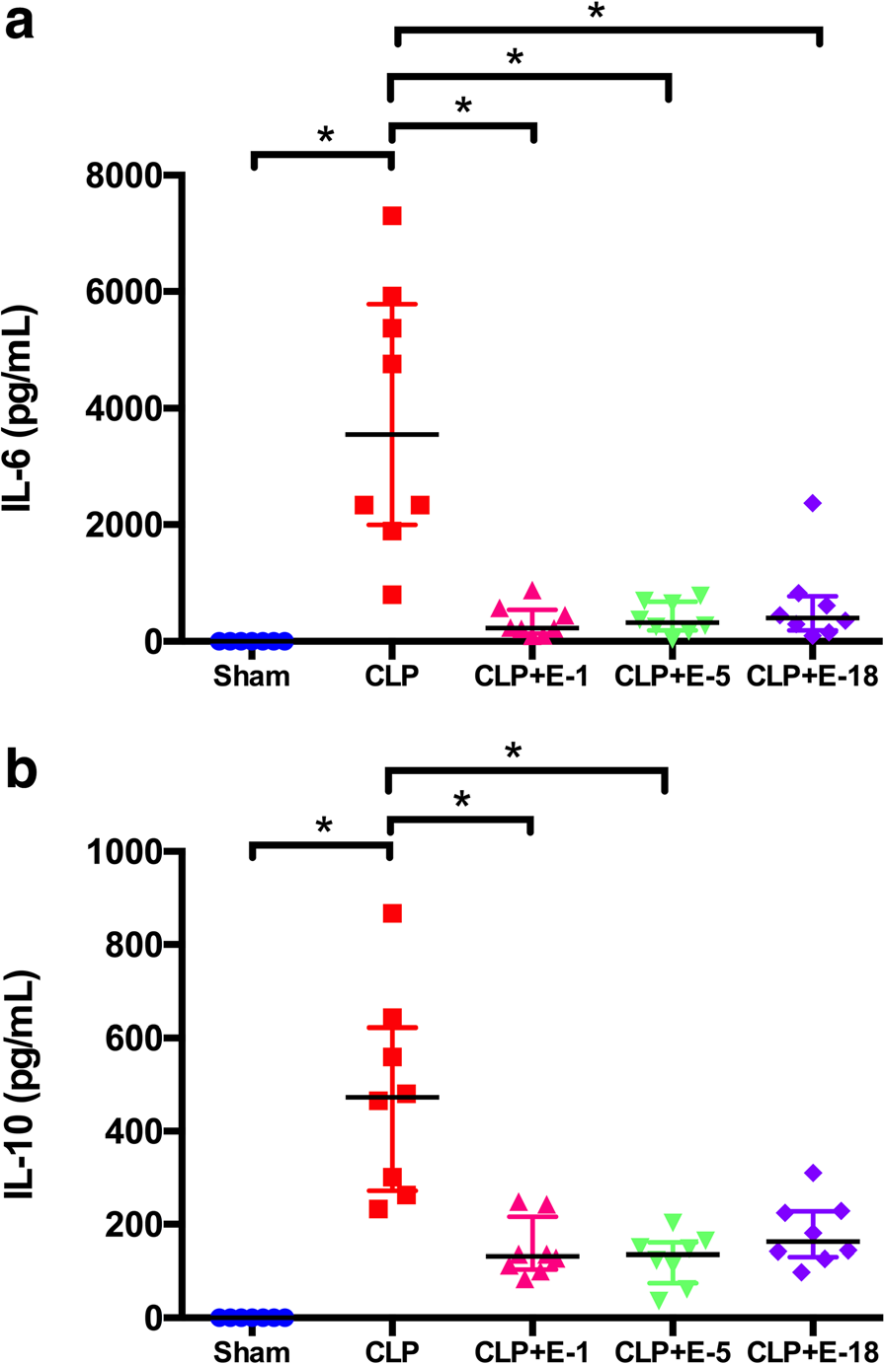
Assessment of circulatory pro-inflammatory/anti-inflammatory cytokine levels of IL-6 (**a**), and IL-10 (**b**) as measured by ELISA. Data are expressed as concentration (pg.ml^−1^); *black lines* indicate medians ± interquartile range (in color). Sham, *n* = 7; all other groups, *n* = 8. *p < 0.05.*CLP* cecal ligation and puncture, *E*-*1* esmolol 1 mg.kg^−1^.h^−1^, *E*-*5* esmolol 5 mg.kg^−1^.h^−1^, *E*-*18* esmolol 18 mg.kg^−1^.h^−1^

Compared to animals in the sham group, CLP was associated with increased plasma levels of IL-6 and IL-10. Addition of esmolol at all tested doses in animals that underwent CLP resulted in a decrease in plasma IL-6 (Fig. 2).

### Effect of different doses of esmolol on CLP-induced inflammatory pathways in cardiac and vascular tissues

**Fig. 3:**
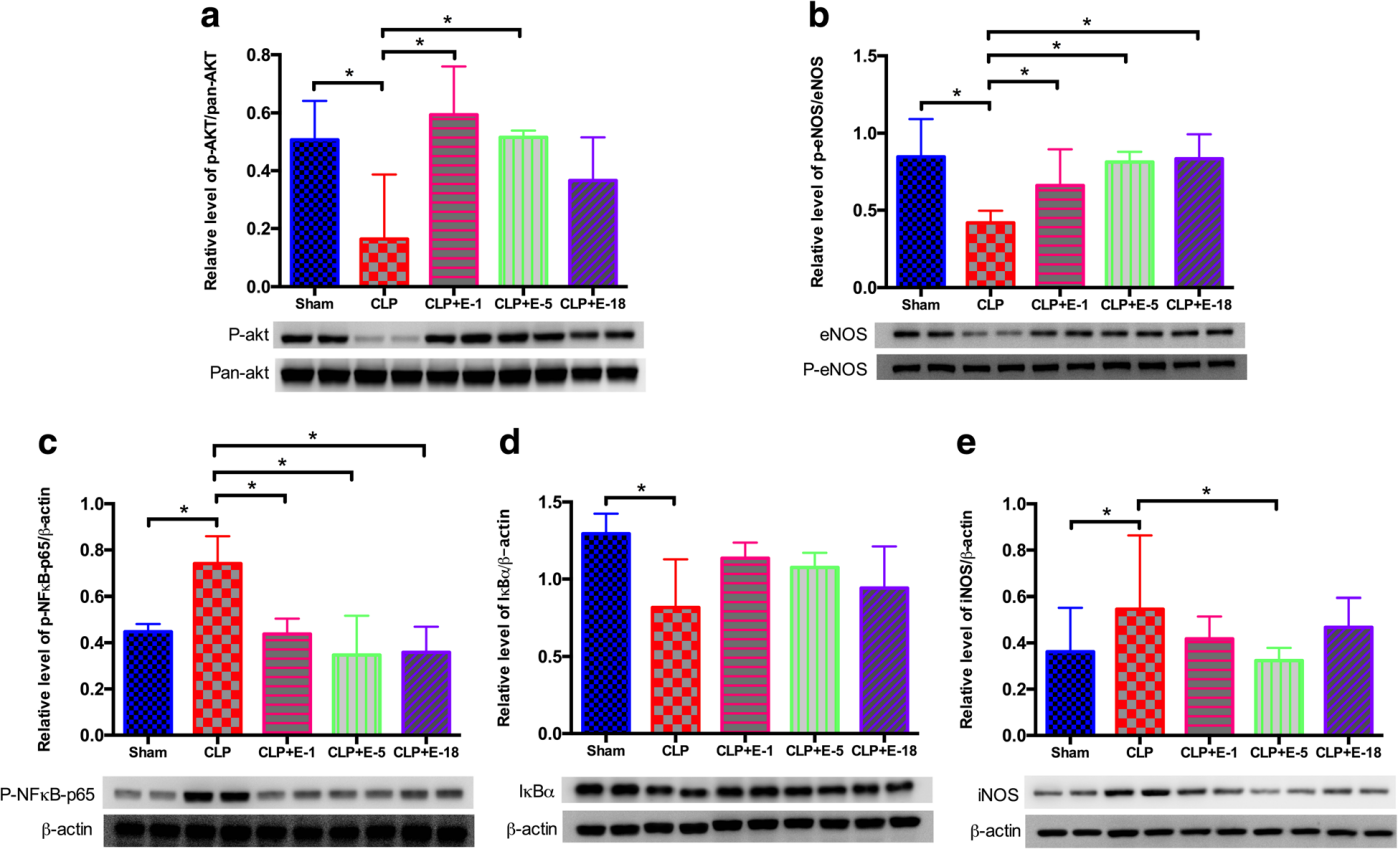
Western blot analysis of protein expression in the heart. Western blots revealing phosphorylated AKT (p-AKT) (**a**), phosphorylated endothelial nitric oxide synthase (p-eNOS) (**b**), nuclear factor - κB (NF-κB) (**c**), nuclear factor of kappa light polypeptide gene enhancer in B-cells inhibitor, alpha (IκBα) (**d**) and inducible nitric oxide synthase (iNOS) (**e**). Proteins were obtained from heart lysates (*n* = 8) prepared from all experimental rat groups. A typical western blot is shown below each *histogram*. Densitometric analysis (*n* = 8) was used to calculate the normalized protein ratio. Data are expressed as median ± interquartile range. *Upper edges* of *error bars* represent the 75th percentile in each group. *p < 0.05. *CLP* cecal ligation and puncture, *E*-*1* esmolol 1 mg.kg^−1^.h^−1^, *E*-*5* esmolol 5 mg.kg^−1^.h^−1^, *E*-*18* esmolol 18 mg.kg^−1^.h^−1^

**Fig. 4:**
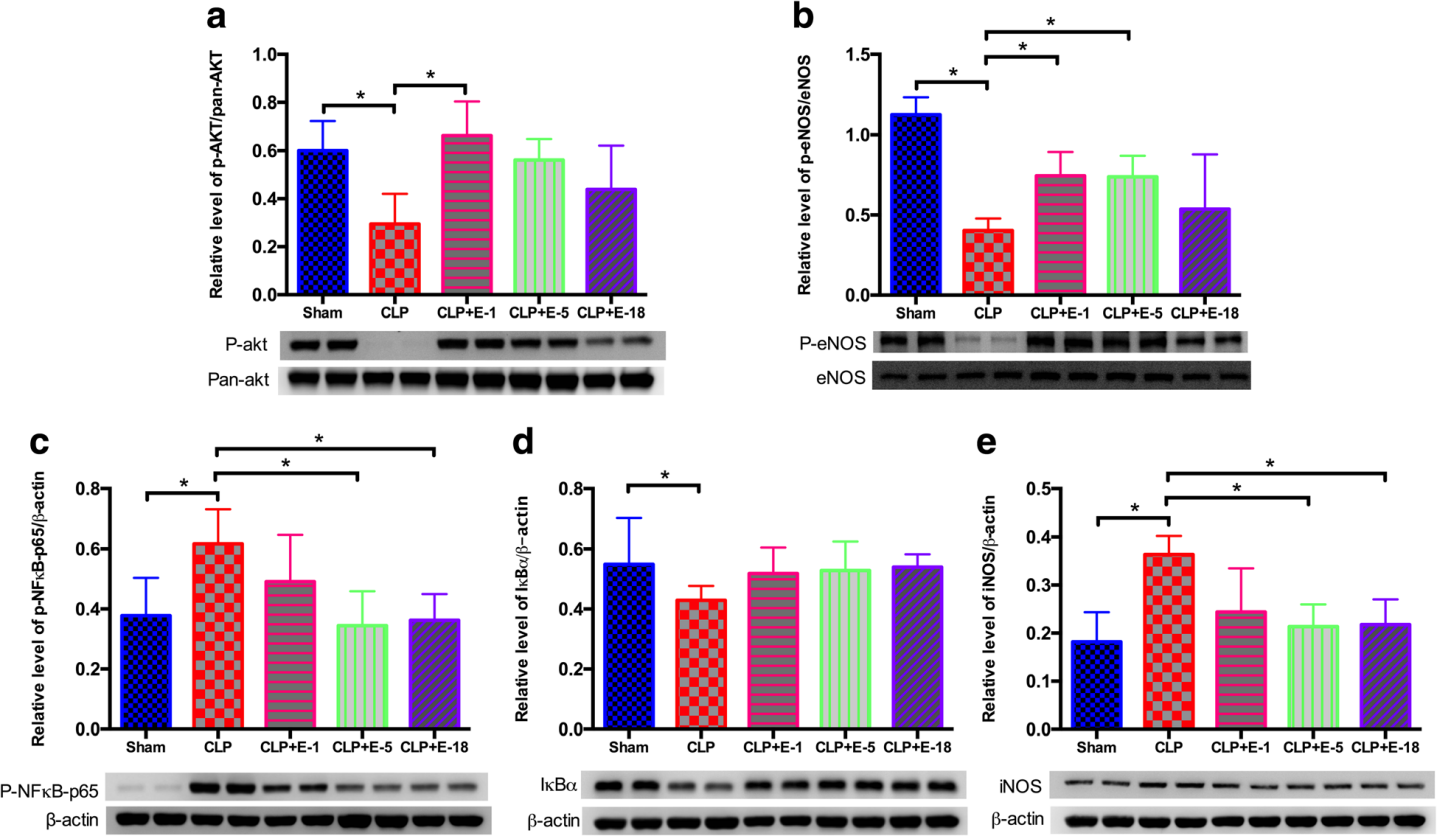
Western blot analysis of protein expression in the thoracic aorta. Western blots revealing phosphorylated AKT (p-AKT) (**a**), phosphorylated endothelial nitric oxide synthase (p-eNOS) (**b**), nuclear factor-κB (NF-κB) (**c**), nuclear factor of kappa light polypeptide gene enhancer in B-cells inhibitor, alpha (IκBα) (**d**) and inducible nitric oxide synthase (iNOS) (**e**). Proteins were obtained from thoracic aorta lysates (*n* = 8) prepared from all experimental rat groups. A typical western blot is shown below each *histogram*. Densitometric analysis (*n* = 8) was used to calculate the normalized protein ratio. Data are expressed as median ± interquartile range. Upper edges of *error bars* represent the 75th percentile in each group. *p < 0.05. *CLP* cecal ligation and puncture, *E*-*1* esmolol 1 mg.kg^−1^.h^−1^, *E*-*5* esmolol 5 mg.kg^−1^.h^−1^, *E*-*18* esmolol 18 mg.kg^−1^.h^−1^

In both the heart and thoracic aorta, CLP was associated with a significant increase in the expression levels of phosphorylated nuclear factor-κB (p-NF-κB), inducible nitric oxide synthase (iNOS) and degradation of nuclear factor of kappa light polypeptide gene enhancer in B-cells inhibitor (IκBα) compared to animals in the sham group, whereas phosphorylated protein kinase B (p-AKT)/pan-AKT ratio and phosphorylated endothelial nitric oxide synthase (p-eNOS)/eNOS ratio were decreased in animals that underwent CLP compared to the sham group (Figs. 3 and 4).

#### Cardiac tissue

Compared to the CLP group, the most complete anti-inflammatory pattern was observed in the CLP + E-5 group of animals. Thus, addition of esmolol at 5 mg.kg^−1^.h^−1^ improved the p-AKT/pan-AKT ratio; increased the p-eNOS/eNOS ratio and decreased the expression levels of p-NF-κB and iNOS (Fig. 3).

#### Thoracic aorta

Changes in the thoracic aorta were similar to those observed in cardiac tissue. Compared to CLP alone, addition of esmolol at 5 mg.kg^−1^.h^−1^ improved the p-AKT/pan-AKT ratio, the p-eNOS/eNOS ratio and decreased the expression levels of p-NF-κB and iNOS.Degradation of IκBα was not influenced by esmolol addition at any dose in cardiac tissue or the thoracic aorta (Fig. 4).

### Adrenergic modulation after infusion of different doses of esmolol

Septic shock was associated with downregulation of α1-adrenoreceptor mRNA in the thoracic aorta (Additional file 2: Figure S2 A1). There was no change in α1-adrenoreceptor mRNA expression induced by the addition of esmolol at any dose (Additional file 2: Figure S2 A-2). Expression of β1-adrenoreceptor and β2-adrenoreceptor mRNA in the heart was unchanged by septic shock (Additional file 2: Figure S2 B1 and C1). Likewise, addition of esmolol at any dose did not induce any change in β1-adrenoreceptor and β2-adrenoreceptor mRNA expression (Additional file 2: Figure S2 B2 and C2).

## Discussion

The main findings of the present study are that, in experimental septic shock, a selective β1-blocker such as esmolol, at a dose that does not decrease heart rate, still improved myocardial function and vasoreactivity. These benefits were associated with downregulation of inflammatory pathways at both the systemic and tissue levels.

### Model characteristics

All animals were resuscitated 4 hours after surgery with adapted antibiotics and fluids in order to mimic the setting usually observed at the bedside. To avoid any interference with the adrenergic system, catecholamines were not administered in this study. In this model, all animals that underwent CLP displayed the typical pattern of septic shock, including hypotension, low cardiac output, hyperlactatemia and vascular hyporesponsiveness to catecholamines, as in our previous study [14, 18]. Moreover, low concentrations of isoflurane were deliberately used to minimize the impact of anesthesia on cardiac and vascular functions [19].

### Effects of esmolol in animals with septic shock

When compared to CLP, the present results demonstrate that low doses of esmolol at 5 and 1 mg.kg^−1^.h^−1^, which did not induce a reduction in heart rate, also displayed beneficial effects on cardiovascular function, including increases in cardiac output, mean arterial pressure and vasoreactivity. While high doses of esmolol have consistently been associated with non-deleterious effects on cardiac and vascular functions in small-animal models of sepsis, the results are more contested in large-animal models [8, 9, 11, 13]. For example, administration of high doses of esmolol in septic pigs has been shown to systematically induce a significant decrease or a trend toward a decrease in the cardiac index [20, 21].

Similarly, in humans, several teams report the efficacy of esmolol, titrated to lower heart rate, in patients with septic shock and tachycardia [10, 15, 16]. For example, Morelli et al., in the JAMA study, demonstrated efficient reduction in heart rate, but also observed a decrease in cardiac output in the first 24 hours in the esmolol group compared to placebo, even though tissue perfusion appeared to be preserved [10]. Similar to our results on vascular reactivity, Morelli et al. also recently found that reduction in heart rate in patients with septic shock treated by esmolol induced an improvement in vascular tone related to a decrease in arterial elastance [16]. In all of these experimental or clinical studies, the reduction in heart rate was achieved after excluding the compensatory nature of tachycardia by adapted fluid resuscitation. Finally, lower doses of esmolol may prove valuable in hemodynamically instable patients in whom the preload independency is difficult to achieve.

### Modulation of inflammation by low doses of esmolol

Findings herein demonstrate that esmolol at low doses still improved cardiovascular function in experimental septic shock without any impact on heart rate, notably through modulation of inflammatory pathways. In keeping with previously published studies of esmolol, reduction in heart rate by ivabradine in an experimental model of septic shock is not associated with a significant decrease in cardiac output, mean arterial pressure or increase in lactate level [8, 10, 11, 14, 15]. One potential explanation is that isolated reduction in heart rate may be associated with economized cardiac function and maintenance of hemodynamic stability. However, in the same experimental study, it was also found that, in contrast to β-blockers, isolated reduction in heart rate by ivabradine did not induce any change in the most commonly assessed inflammatory pathways in rats with septic shock [14]. Thus, it could be argued that the correction of elevated heart rate, even if crucial (particularly when tachycardia is prolonged in time), is not the only expected endpoint with the prescription of β1-blockers during septic shock [22].

At the initial stages, patients with septic shock have an exaggerated pro-inflammatory and anti-inflammatory response, the severity of which is related to outcome [23]. In experimental models of septic shock, including the present study, addition of esmolol at all doses was systematically associated with modulation of inflammation at both systemic and tissue levels [8, 9, 11, 13]. Similar to Ackland et al., we also observed global downregulation of both anti-inflammatory and pro-inflammatory cytokines by esmolol in our study [13]. This modulation of inflammation could also account for the findings in the studies of Fuchs et al. and Macchia et al., in which chronic administration of β-blockers was associated with an improved survival rate in patients who subsequently developed septic shock [24, 25]. Consequently, β1-blockers may act at two levels: hemodynamically by way of reduction in heart rate, and in an inflammatory manner through their direct anti-inflammatory properties [26].

### Modulation of adrenergic receptor mRNA expression with esmolol

Septic shock is most often associated with downregulation of vascular α1-adrenoreceptor expression [27, 28]. This downregulation is likely a consequence of a massive NF-κB activation-induced release of pro-inflammatory cytokines generated by sepsis [29]. In our previous study, addition of esmolol 18 mg.kg^−1^.h^−1^ was associated with significant improvement in the mRNA level of α1-adrenoreceptor [11]. However, we were unable to confirm this finding in the present study. One possible explanation is that in order to be as accurate and reliable as possible, three housekeeping genes were used in the present study to normalize the results versus only one in the previous study. Indeed, housekeeping gene expression can vary considerably, as reported in numerous studies [30, 31].

No change in β1-adrenoreceptor and β2-adrenoreceptor mRNA expression was observed in the heart in animals with sepsis in the current study. Only one experimental study has suggested that septic shock is associated with reduction in β1-adrenoreceptor mRNA and protein levels in heart tissue in the late phase [32]. In addition, it could also be hypothesized that the total amount of adrenoreceptors produced does not significantly differ during septic shock and consequently that mRNA quantification may not be the appropriate measure. Indeed, adrenoreceptor desensitization includes several other processes, including phosphorylation, catecholamine-induced internalization and degradation via a lysosomal pathway [33, 34]. Investigating these processes may prove of interest in future studies.

### Study limitations

Despite massive fluid administration 10 ml.kg^−1^.h^−1^, we cannot rule out the possibility that our CLP model displayed some degree of hypovolemia considering that in animals that underwent CLP, cardiac output was markedly decreased and that the ejection fraction tended to increase. In the study of Rudiger et al., the decline in stroke volume and left ventricular end-diastolic volume compared to animals in the sham group was attenuated by an additional 25 ml.kg^−1^ body weight of fluid boluses of hetastarch at 6 hours and 10 ml.kg^−1^ at 24 hours [35]. It is likely that additional volume fluid loading in our mode, would have been beneficial to hemodynamic recovery. At the bedside, such a clinical pattern should be corrected by individually adapted fluid and vasopressor treatment, even though fluid management remains extremely challenging in patients with septic shock [36]. Consequently, while reduction in heart rate is counterintuitive in instances of hypovolemia, on the contrary, a lower dose of esmolol may appear more appropriate in patients with septic shock. In the present design, we attempted a minimally invasive strategy for the assessment of cardiac function by echocardiography. As a result, we were not able to assess intrinsic myocardial inotropism and thus, confirm the observed improvement in the esmolol groups. Finally, it would have been of particular interest to investigate the mortality rate in each group in a specific survival sub-study.

## Conclusion

During experimental septic shock, addition of low doses of esmolol, which do not induce reduction in heart rate, is associated with in vivo hemodynamic improvements and better ex vivo vasoreactivity. These benefits appear to be related to inflammatory modulation at both the systemic and tissue levels. These findings, which necessarily need to be confirmed, offer new insights into the approach to prescribing esmolol during septic shock and in the design of future clinical trials of esmolol use during septic shock.

## Additional files

Additional file 1: Methodology for (1) shock model, (2) echocardiography, (3) vascular reactivity and (4) RNA extraction and quantitative reverse transcriptase-polymerase chain reaction. (DOCX 23 kb)
Additional file 2: Figure S1. Effect of different doses of esmolol on heart rate in rats with sepsis. **Figure S2.** Messenger RNA (mRNA) of α1-adrenoreceptors (**A**), β1-adrenoreceptors (**B**) and β2-adrenoreceptors (**C**) in the thoracic aorta or heart. (DOCX 20140 kb)

## Abbreviations

Ach: acetylcholine
AKT: protein kinase B
CLP: cecal ligation and puncture
ELISA: enzyme-linked immunosorbent assay
eNOS: endothelial nitric oxide synthase
IgG,: immunoglobulin G
IL-10: interleukin 10
IL-6: interleukin 6
iNOS: inducible nitric oxide synthase
IQR: interquartile range
IκBα: nuclear factor of kappa light polypeptide gene enhancer in B-cells inhibitor
mRNA: messenger ribonucleic acid
NF-κB: nuclear factor-κB
p-AKT: phosphorylated protein kinase B
p-eNOS: phosphorylated endothelial nitric oxide synthase
Phe: phenylephrine
p-NF-κB: phosphorylated nuclear factor-κB
RNA: ribonucleic acid
SMA: small mesenteric arteries
