# Supplementary material for: Effects of low doses of esmolol on cardiac and vascular function in experimental septic shock
Source: Crit Care. 2016 Dec 21;20:407. doi: 10.1186/s13054-016-1580-2 (PMC5175382; doi:10.1186/s13054-016-1580-2)
Supplement: Additional file 1: — Methodology for (1) shock model, (2) echocardiography, (3) vascular reactivity and (4) RNA extraction and quantitative reverse transcriptase-polymerase chain reaction. (DOCX 23 kb) [file 13054_2016_1580_MOESM1_ESM.docx]

*Shock model: CLP (cecal ligation and puncture).* Briefly, in isoflurane-anesthetized rats (induction: isoflurane 3.5% and maintenance: isoflurane 2%), a laparotomy was performed after which the cecum was ligated just below the ileo-cecal valve and punctured with an 18-gauge needle, followed by extrusion of cecal contents. For sham-operated rats, the cecum was exteriorized without ligation or puncture. The cecum was thereafter re-inserted into the abdomen and the incision closed by suture. A catheter was concurrently inserted in the left external jugular vein and tunneled subcutaneously to the back of the neck where it was attached to a swivel device. Four hours after the experiment, all rats received a continuous infusion of saline, analgesic (nalbuphin 0.2 mg.kg^-1^.h^-1^) and anti-infective therapy (imipenem and cilastatin sodium 10 mg.kg^-1^). Total infusion rate was fixed at 10 ml.kg^-1^.h^-1^.

*Echocardiography procedure.* Rats were anesthetized with 1.5% isoflurane using 100% oxygen for echocardiographic assessment (12 mHz linear-array probe at an image depth of two centimeters on a HP Sonos 5500 Imaging System, Philips Medical Systems, Andover, MA, USA). Ascending aortic diameter (AD) was obtained at systole from the left parasternal long axis view. Ejection fraction (EF) was defined from left ventricular end-systolic and end-diastolic dimensions measured in short axis view at the level of the papillary muscles in M mode. Aortic velocity time integrate (VTI) was defined from the apical five-chamber view. Heart Rate (HR) was calculated from the time interval period (T) between two maximum peak velocities (HR=60/T). Stroke volume (SV) and cardiac output (CO) were calculated with the following formulas: SV=VTI×AD^2^/4 and CO= HR×SV, respectively. All measurements were averaged on three consecutive cardiac cycles. Mean arterial pressure was measured by a blood pressure transducer (Emka, Paris, France) through the left carotid artery. Lactate concentrations were determined using a Statstrip Lactate Xpress Meter (Nova Biomedical, Flintshire, UK)

*Vascular Reactivity Procedure.* Vascular reactivity of thoracic aorta and small mesenteric artery rings (radius 200-400μm) was studied on a wire myograph (Danish Myo Technology, Arhus, Denmark) as previously described (1). The experiments were performed at 37°C in a physiological salt solution (PSS) with the following composition (in mM): 119 NaCl, 4.7 KCl, 14.9 NaHCO_3_, 1.2 MgSO_4_7H_2_O, 2.5 CaCl_2_, 1.18 KH_2_PO_4_, and 5.5 glucose, continuously bubbled with 95% O_2_ and 5% CO_2_. After a 20-min equilibration period under optimal passive tension, two successive contractions in response to the combination of KCl depolarization (80 mM) and phenylephrine (Phe) (10μM) (Sigma-Aldrich, Saint Quentin Fallavier, France) were used to test the maximal contractile capacity of the vessels. Following a 20-min washout period, concentration-response curves to Phe were elicited by cumulative administration of this vasoconstrictor agonist (1 nM to 10μM) in order to determine whether the same concentration produced an equal level of contraction in the different groups. To study endothelium-dependent relaxation, small mesenteric rings with functional endothelium were precontracted with Phe (10µM) and then exposed to increasing incremental concentrations of acetylcholine (Ach) (1nM to 10 µM; Sigma, St Louis, MO, USA).

*α1-, β1- and β2-adrenoreceptor RNA Extraction and Quantitative Reverse Transcriptase-Polymerase Chain Reaction.* Total RNA was reverse-transcribed to complementary DNA (cDNA) using the iScript™ cDNA synthesis kit (Biorad, Marnes-la-Coquette, France). cDNA obtained from the RT reaction was subjected to quantitative PCR using iQ™ SYBR^®^ Green Supermix (Biorad, Marnes-la-Coquette, France). Primers were obtained from Quiagen (Quiagen, Courtaboeuf Cedex, France). The primer concentrations were optimized according to the manufacturer’s guidelines. A gradient temperature from 56°C to 62°C was tested for each primer in order to establish the optimized annealing temperature. Annealing at 62°C was chosen for all primers for the PCR reactions with the following reaction variables: polymerase activation and cDNA denaturation at 95°C for three minutes, followed by denaturation at 95°C for 10 seconds, annealing at 62 °C for 45 seconds and extension at 72°C for one minute for 50 amplification cycles. Each sample was determined in duplicate. To determine primer efficiency, a standard curve for each gene was created using RNA isolated from the Sham group. Isolated RNA was reverse-transcribed, and dilution series of cDNA ranging from 1 to 10^-4^ were subjected to real-time PCR. The obtained threshold cycle values were plotted against the dilution factor to create a standard curve. Primer efficiency was then calculated from the standard curve. Expressions of α1-, β1- and β2-adrenoreceptor messenger RNA (mRNA) in heart and in thoracic aorta respectively as well as four housekeeping genes (beta-actin, RPS29, GAPDH, HGPRT) mRNA were measured. The three most stable housekeeping genes (beta-actin, RPS29, HGPRT) were chosen to normalize the expression of α1-, β1-, β2-adrenoreceptors, after which the expression of α1-, β1-, β2-adrenoreceptors in other groups was normalized to the Sham group.

**Reference**

1. Kimmoun A, Ducrocq N, Sennoun N, Issa K, Strub C, Escanye JM, et al. Efficient extra- and intracellular alkalinization improves cardiovascular functions in severe lactic acidosis induced by hemorrhagic shock. Anesthesiology. 2014;120:926-34.
