# Supplementary material for: Effects of low doses of esmolol on cardiac and vascular function in experimental septic shock
Source: Crit Care. 2016 Dec 21;20:407. doi: 10.1186/s13054-016-1580-2 (PMC5175382; doi:10.1186/s13054-016-1580-2)
Supplement: Additional file 2: Figure S1. — Effect of different doses of esmolol on heart rate in rats with sepsis. Figure S2. Messenger RNA (mRNA) of α1-adrenoreceptors (A), β1-adrenoreceptors (B) and β2-adrenoreceptors (C) in the thoracic aorta or heart. (DOCX 20140 kb) [file 13054_2016_1580_MOESM2_ESM.docx]

**Figure S1.** **Effect of different doses of esmolol on heart rate in septic rats.** Esmolol was infused four hours after onset of CLP at 18 and 5 mg.kg^-1^.h^-1^. Heart rate was measured by echocardiography at 18 hours after onset of CLP. Data are expressed as median and interquartile range. CLP, n=8; CLP+E-18, n=4; CLP+E-5, n=4. Data of the CLP group were extracted from our previous study on ivabradine (1). CPL: Cecal Puncture and Ligation.

*Significant difference compared with the CLP group (P<0.05).


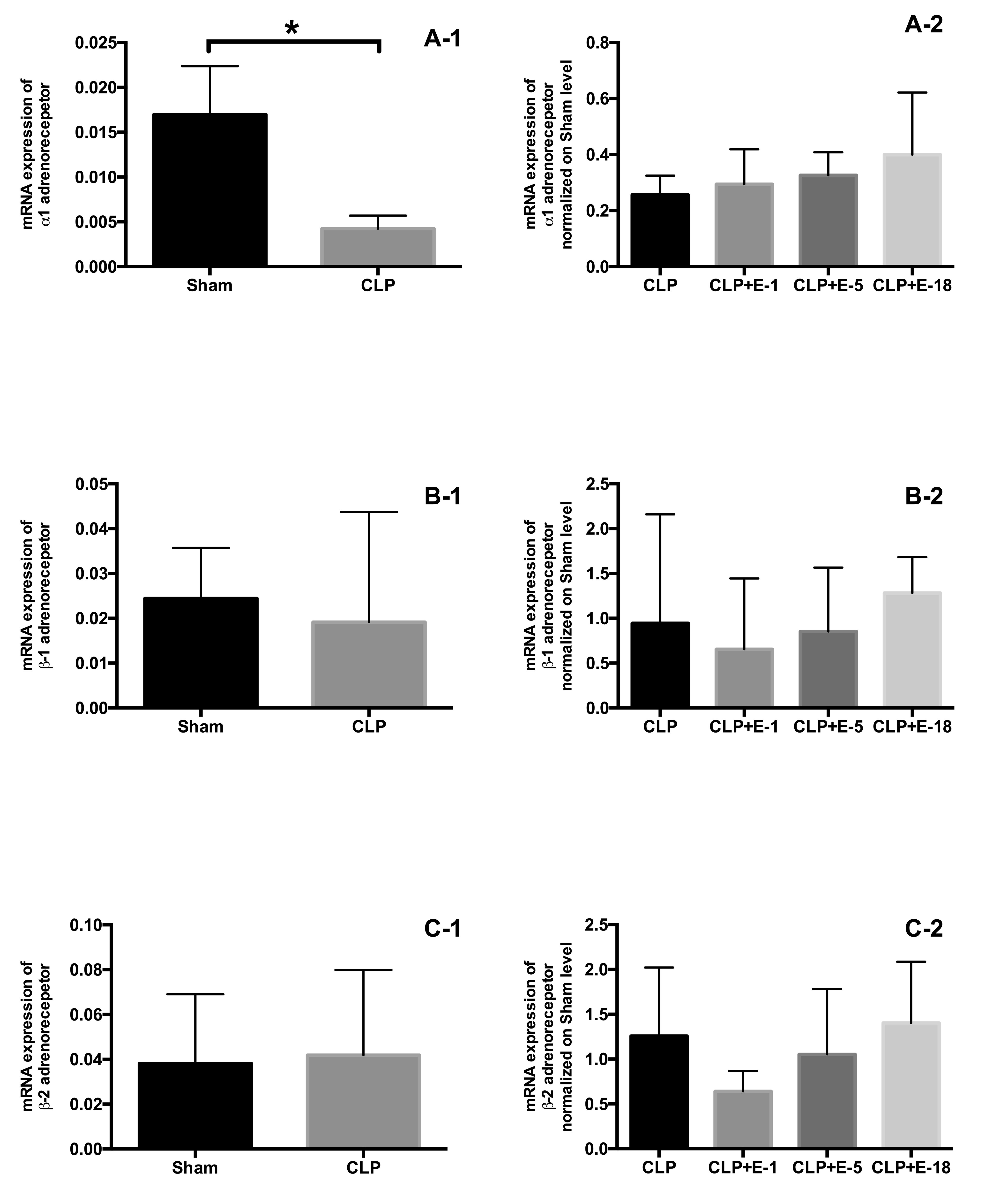


**Figure S2. Messenger RNA (mRNA) of (A) α1 (B) β1 and (C) β2 -adrenoreceptors in thoracic aorta or heart.** In A-1, B-1 and C-1, results were normalized to three housekeeping genes (β-actin, GAPDH and RP29S). In A-2, B-2 and C-2, results were first normalized to the three housekeeping genes and thereafter to sham expression, which was set at 1. Data are expressed as median ± interquartile range deviation. Upper edges of error bars represent the 75th percentile in each group. β1 and β2-adrenoreceptor mRNA in heart: CLP, n=7; all other groups, n=8. α1-adrenoreceptor mRNA in thoracic aorta: Sham, n=7; CLP+E-1, n=7; all other groups, n=8. * p < 0.05. CLP = cecal ligation and puncture.

Reference

1. Wei C, Kattani NA, Louis H, Albuisson E, Levy B, Kimmoun A. If Channel Inhibition with Ivabradine Does not Improve Cardiac and Vascular Function in Experimental Septic Shock. Shock. 2016;46:297-303.
